# MDC1 depletion promotes cisplatin induced cell death in cervical cancer cells

**DOI:** 10.1186/s13104-020-04996-5

**Published:** 2020-03-11

**Authors:** Neeru Singh, Rashmi Bhakuni, Dimple Chhabria, Sivapriya Kirubakaran

**Affiliations:** 1 Indian Institute of Technology Gandhinagar, Palaj Campus, Gandhinagar, Gujarat 382355 India; 2 Biological Engineering, Indian Institute of Technology Gandhinagar, Palaj, Gujarat 382355 India

**Keywords:** Cisplatin, Cervical cancer, DNA damage repair, Ataxia telangiectasia mutated, MDC1, Apoptosis

## Abstract

**Objective:**

Cisplatin, the most common chemotherapeutic drug for the treatment of advanced stage cervical cancers has limitations in terms of drugs resistance observed in patients partly due to functional DNA damage repair (DDR) processes in the cell. Mediator of DNA damage checkpoint 1 (MDC1) is an important protein in the Ataxia telangiectasia mutated (ATM) mediated double stranded DNA break (DSB) repair pathway. In this regard, we investigated the effect of MDC1 change in expression on the cisplatin sensitivity in cervical cancer cells.

**Results:**

Through modulation of MDC1 expression in the cervical cancer cell lines; Hela, SiHa and Caski, we found that all the three cell lines silenced for MDC1 exhibited higher sensitivity to cisplatin treatment with inefficiency in accumulation of p γH2AX, Ser 139 foci and increased accumulation of pChk2 Thr 68 at the damaged chromatin followed by enhanced apoptosis. Further, we observed the increased p53 Ser 15 phosphorylation in the MDC1 depleted cells. Our studies suggest that MDC1 expression could be a key determinant in cervical cancer prognosis and its depletion in combination with cisplatin has the potential to be explored for the sensitisation of chemo-resistant cervical cancer cells.

## Introduction

Cervical cancer is amongst the commonly diagnosed cancers in women, with a significant proportion of patients treated with cisplatin based chemo-radiotherapy (CRT) [1]. Genomic instability and high mutation rates exhibited in cervical cancers have been reported partly due to the persistent infection with human papillomavirus (HPV) and inactivation of important tumour suppressor genes [2, 3]. Few studies report that HPV proteins activate the ATM-Chk2 based DNA damage response pathway implicating its importance for viral genome amplification [4]. Thus, down-regulation of ATM pathway can prove to be an effective therapeutic approach towards blocking the spread of HPV infections. However, the dejected outcomes including the lack of specific ATM kinase inhibitors [5] have stimulated research efforts to identify molecular characteristics specific to a certain tumour to predict their resistance (via the activation of DDR pathways) to CRT or recommend the utilization of novel targeted therapies [1] as cisplatin resistance remains one of the major concerns and causes of cervical cancer recurrences.

In this regard, MDC1, a mediator protein activated as part of ATM-ChK2 pathway is necessary for proper manifestation of the DDR response [6] and can be a limiting factor affecting prognosis of certain cancers [7]. MDC1 has—N-terminal fork head associated (FHA) domain, central PSTrich) domain and two C-terminal BRCT domains [8]. It regulates intra-S phase, G2/M checkpoints and actively participates in DSBs repair by both homologous recombination (HR) [9] and nonhomologous end joining (NHEJ) pathway [10]. At the site of damaged chromatin, ATM initiates a signalling cascade wherein a damaged chromatin is marked by γH2AX, a Histone 2A variant phosphorylated at Serine 139 residue which is bound by phosphorylated MDC1 [11, 12]. This interaction promotes the recruitment and retention of necessary DDR proteins at the damaged chromatin [13–15] which supports positioning of additional ATM molecules resulting in amplification of DDR signalling along the chromatin length. MDC1 silencing in combination with radiation or chemotherapeutic agents presents a rational strategy for the treatment of ATM proficient cancers, which primarily depend on the ATM-Chk2 pathway activation for the repair of double strand DNA breaks. In the present study, we have shown MDC1 can explored as a therapeutic target for improving cervical cancer prognosis in combination with drugs like cisplatin.

## Main text

### Materials and methods

#### Cell culture

Cervical cancer cell lines HeLa, SiHa and CasKi, were procured from National Centre for Cell Science, Pune. The cells were cultured in DMEM (Thermo Fisher Scientific, Waltham, MA, USA) with 10%FBS, 100 U/ml penicillin, 100 μg/ml streptomycin, and 4 mM l-glutamine and incubated at 37 °C in a CO2incubator. Cisplatin (P4394) was procured form Sigma Aldrich and diluted in 0.45% NaCl saline solution.

#### Antibodies

p-H2AX Ser 139 mAb (2577S), p-p53 Ser15 mAb (9284S), p-Chk2 Thr68 mAb (2661) Anti-rabbit IgG HRP linked (7074), Anti-mouse IgG HRP linked secondary antibody (7076), Anti-IgG (H+L) F(ab′)2 Alexa Fluor 488 (4412) and Anti-rabbit IgG (H+L) F(ab′)2 Alexa Fluor 555 (Cat. 4413) were obtained from Cell Signalling Technology. MDC1 mouse mAb (1–50) (ab50003) from Abcam and anti human β-Actin (Cat No. SC47778HRP) was purchased form Santa Cruz Biotechnology.

#### Stable cell line generation

GIPZ lentiviral MDC1 shRNA (Dharmacon, Inc.) and pCDNA3 MDC1(a kind gift from Prof. Michel Goldberg, Hebrew University, Jerusalem) (Additional file 1: Figure S1 and Additional file 2: Figure S2) were used to generate the stable cervical cancer cell lines using Lipofectamine (Thermo fisher scientific). The stable cell lines were rigorously selected and maintained in 2.5 μg/ml puromycin and 400 μg/ml G418 for MDC1 knockdown and MDC1 overexpression, respectively.

#### Cell viability assay

Cell viability assay was done using Cell Titer-Glo luminescent assay kit (Promega, USA) based on quantitation of the ATP. The cells were seeded at a count of 2000 cells per well in a 96 well plate and cisplatin treatment was given to the cells at different concentrations of 5, 10 and 20 μM for 72 h and luminescence reading (Envision, Perkin Elmer).

#### Clonogenic survival assays

Cells were treated with cisplatin (10 μM) for 72 h after which they were trypsinized and seeded in 6 cm dishes in triplicate at a count of 1000 cells per plate. After 14 days of incubation the colonies were fixed with methanol and stained with crystal violet (0.5% crystal violet in 20% Methanol, Sigma) and the colony numbers counted. The colonies with ≥ 50 cell count as observed under a stereo microscope were considered for the analysis. Number of colonies derived from the untreated control cells was set as 100% (reference) for comparison. The surviving fraction was calculated by dividing the average number of visible colonies in treated versus untreated dishes.

#### Western blotting

Cells were seeded in 6 cm dishes (0.8 × 106/dish) and grown overnight. After treatment with cisplatin cells were lysed in ice-cold RIPA buffer (Sigma, R0278). The soluble fractions of cell lysate isolated by centrifugation at 13,000 rpm for 10 min in a micro centrifuge at 4 °C. and analysed by SDS-PAGE and western blotting (transfer onto PVDF membrane, Bio-Rad, USA). The membrane was blocked with 5% non-fat milk for 2 h at room temperature incubated with the primary antibody at 4 °C overnight with gentle shaking. Respective secondary antibodies were added the next day and blots were visualised using the Clarity Western ECL luminescent substrate (Cat No. 1705061) according to the manufacturer’s instructions (Bio-Rad Laboratories). Beta-actin was used a loading control.

#### Immunofluorescence assay

Cells were seeded on poly l-lysine coated coverslips treated with cisplatin (10 μM) for 2 h fixed with 4% paraformaldehyde for 15 min, permeabilized with 0.3% Triton X-100/methanol solution for 10 min, blocked with bovine serum albumin/fetal bovine serum for 1 h. Primary antibody incubation was performed overnight at 4 °C. Cells were stained with TRITC labelled secondary antibody (Alexa Fluor 555 conjugate) for ~ 2 h at room temperature. DAPI i.e., 4-6-diamidino-2-phenylindole dihydrochloride (Cat no. NC9524612; VECTASHIELD Antifade Mounting Medium, Fisher Scientific, USA) was used to stain the nuclei.

#### Flow cytometry analysis of apoptosis

Cells were seeded in 6 cm dishes, incubated for 24 h with 10 μM Cisplatin when 80–90% confluent and stained with cyanine 3-conjugated annexin V (AnnexinV-Enzogold) and propidium iodide (PI) using the GFP certified Apoptosis/Necrosis Detection Kit (ENZ-51002, Enzo Biochem, Inc. New York, USA) as per the manufacturer’s recommendation. Each sample was then subjected to analyses by flow cytometry (FCM) using S3e cell sorter (BIO-RAD, Hercules, CA, USA).

#### Statistical analysis

GraphPad Prism software (version 5.01) was used for statistical analysis, and *p* < 0.05 was considered statistically significant (*p < 0.05, **p < 0.01, ***p < 0.001). All the tests were either one-way or two-way analysis of variance (ANOVA) followed by multiple comparison post-test.

### Results

#### Stable cell line generation and effect of cisplatin on MDC1 expression modulated cervical cancer cells

**Fig. 1:**
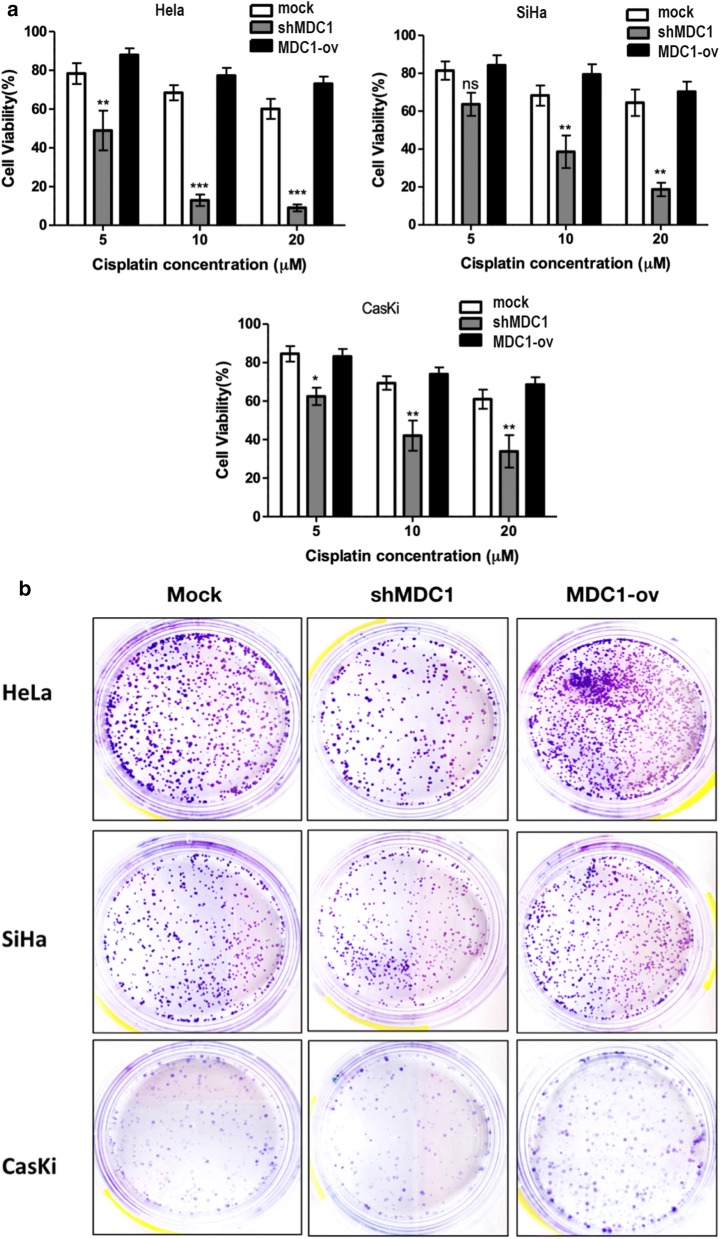

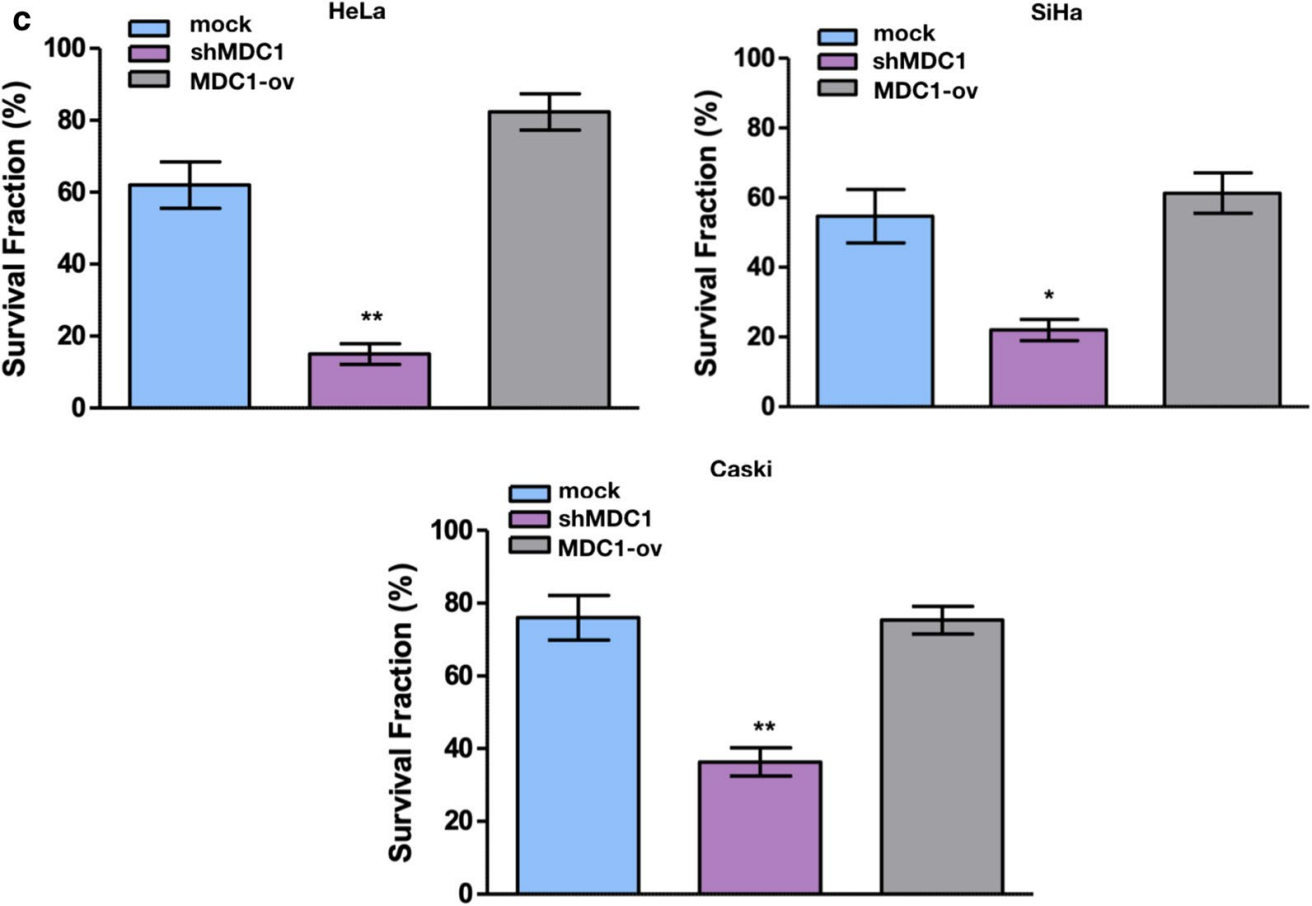
MDC1 silenced cells are sensitive to cisplatin treatment. **a** Effect of increasing dosage of cisplatin after 72 h of exposure on percentage cell viability as evaluated in MDC1 depleted (shMDC1) and overexpressing (MDC1-ov) cell lines. **b** Colony survival assay was done to determine colony formation after treatment with 10 µM cisplatin. **c** Graphical analysis of the colony survival assay using GraphPad Prism 5.0 software

Stable cervical cancer cell lines were generated (Additional file 3: Figure S3). We tested the sensitivity of the cell lines towards cisplatin at four different concentrations i.e. 5, 10, 15 and 20 μM for a duration of 72 h by CTGassay. Cisplatin exposure induced significant reduction in the cell viability in a dose dependent manner in MDC1 silenced cell lines (Fig. 1a).

Further, colony survival assay was performed after cisplatin (10 μM) treatment). Considerably smaller number of surviving colonies were observed in MDC1 knocked down cell lines whereas MDC1 high expression rendered the cell lines insensitive to the drug treatment, evident as maximum number of colonies observed in these cell lines (Fig. 1b and c). The difference between control cells and MDC-ov cells was found to be statistically insignificant in all the three cervical cancer cell lines studied (Additional file 4: Figure S4).

#### MDC1 promotes cisplatin induced γH2AX phosphorylation and foci formation and inhibits ChK2 accumulation

**Fig. 2:**
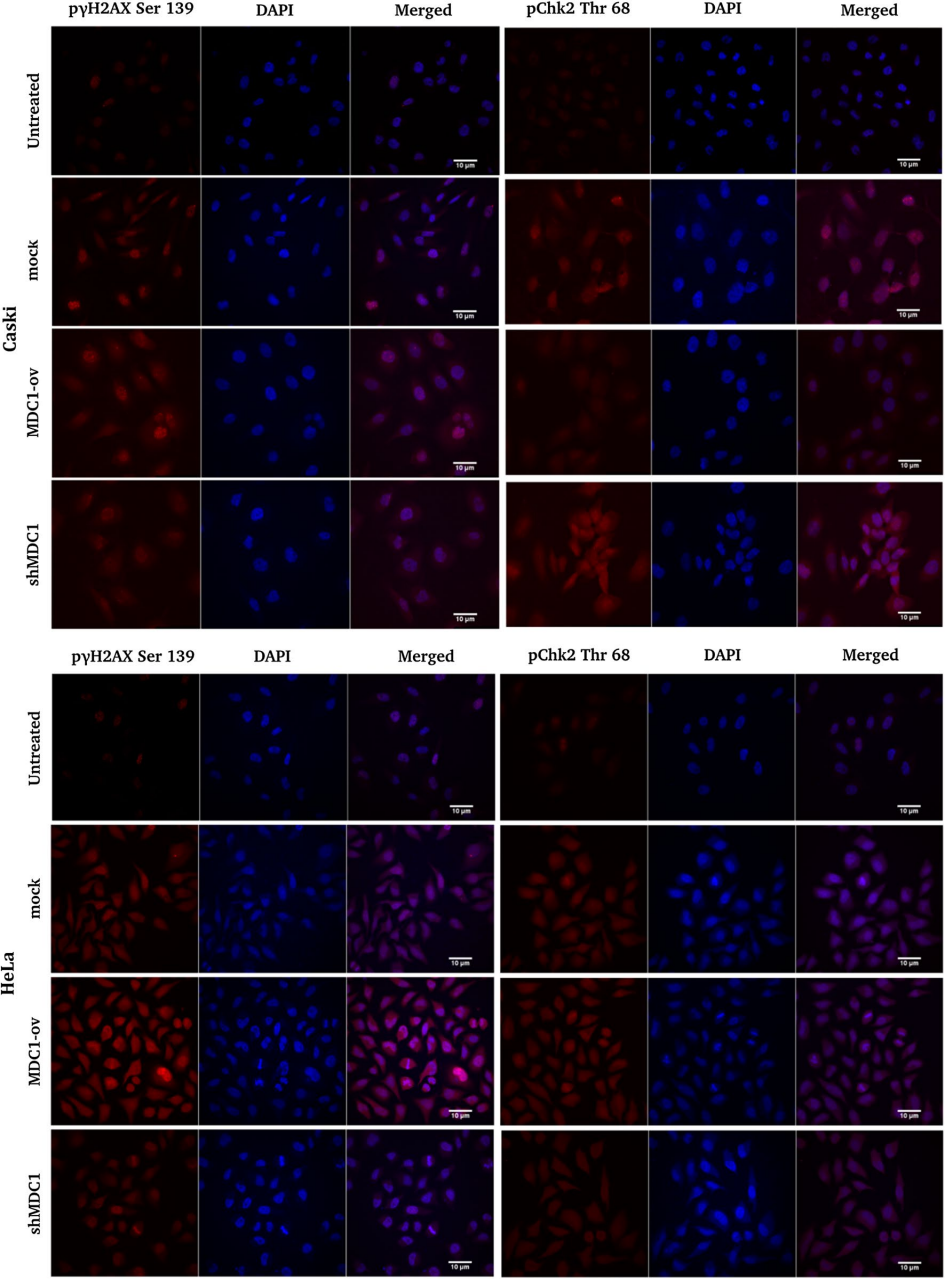

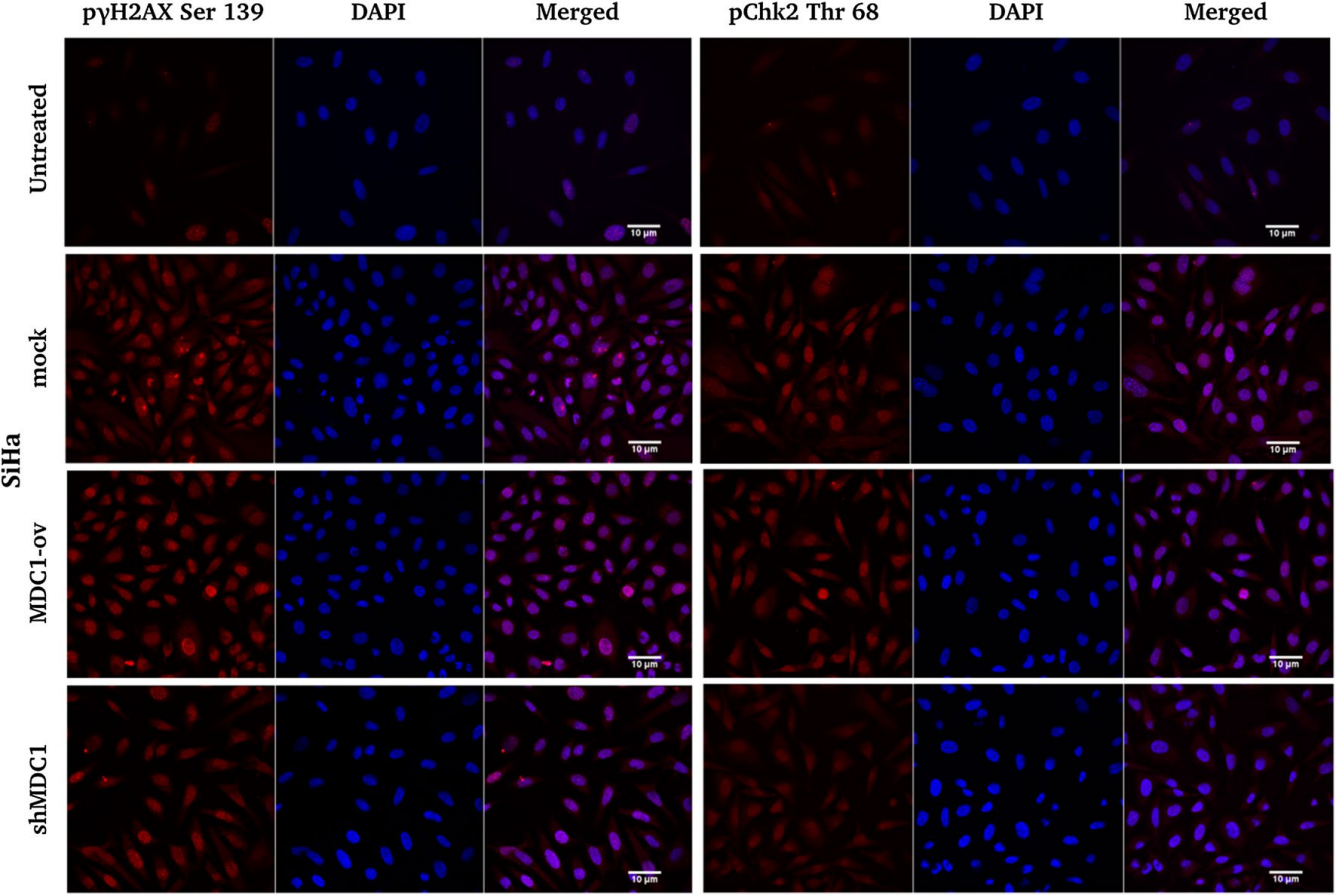
Analysis of the accumulation of γH2AX and ChK2 DDR proteins in response to cisplatin (10 µM) induced DNA damage in stable cervical cancer cell lines. Immunofluorescence studies were done to estimate the γH2AX and ChK2 Thr68 recruitment in MDC1 modulated cervical cancer cell lines exposed to cisplatin treatment. Scale bar: 10 μm

MDC1 regulated γH2AX phosphorylation and interaction is a major signal for the recruitment of DDR proteins to the regions of damaged chromatin [15]. We performed immunofluorescence studies with our cell lines exposed to 2 h of cisplatin treatment and observed significant decline in the γ-H2AX foci formation in MDC1 knocked down cell lines with a concomitant increased accumulation of the same in MDC1 overexpressed cells. On the contrary, the Chk2 accumulation was noticed to be considerably high in MDC1 depleted cell lines supporting p53 stabilization and activation (Fig. 2).

#### Enhanced apoptosis in MDC1 knocked down cells upon cisplatin exposure followed by increased p53 serine 15 phosphorylation

**Fig. 3:**
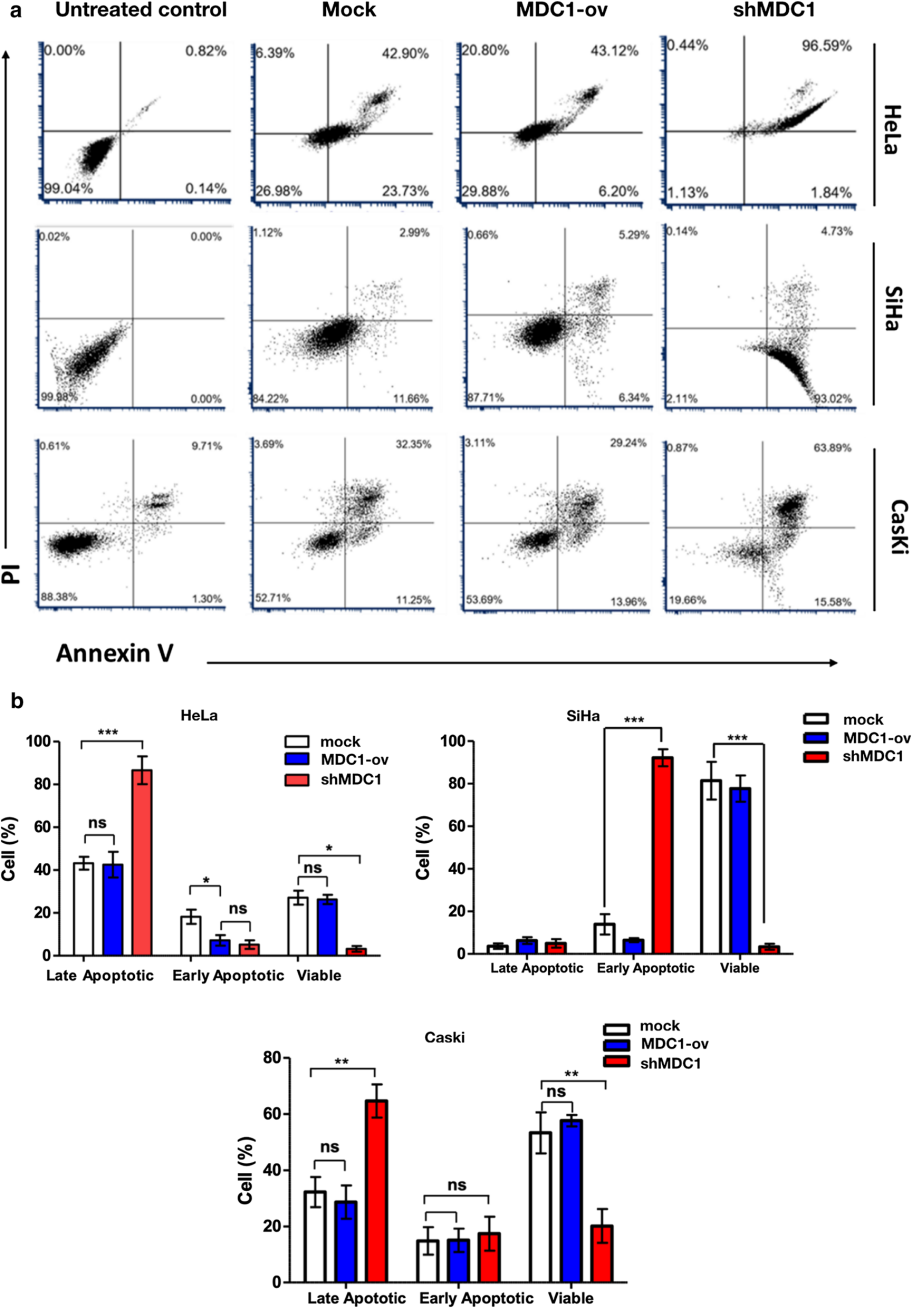

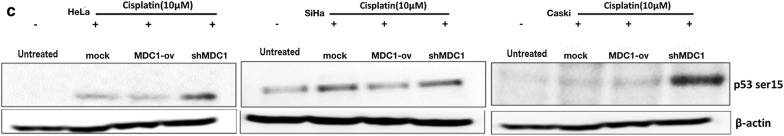
Cisplatin induced cytotoxicity results in higher percentage of cells undergoing apoptosis in MDC1 silenced cell lines. **a** Annexin V and propidium iodide labelling of cells was done after exposure to cisplatin (10 μM) for 24 h and analysis was done using flow cytometry software. **b** Graphical analysis of the apoptosis data using GraphPad Prism 5.0 software. **c** Western blot was performed to check p53 Ser 15 status in the cell lines after exposure to Cisplatin (10 µM) for 24 h. Beta actin was taken as the loading control

To evaluate the apoptotic rate in the cell lines on cisplatin exposure, we performed annexin V and PI labelling after 24 h of treatment and monitored the cells by FCM. Our results showed high cisplatin sensitivity in combination with MDC1 depletion in all the three cervical cancer cell lines (Fig. 3a and b). Next, we analysed the phosphorylation status of p53 at Ser 15 residue by western blotting (Fig. 3c). The p53 function has been reported to be down regulated in case of HPV infected cervical cancer cells because of the overexpression of E6 and E7 oncogenic proteins [16, 17]. Simultaneously, Phosphorylation of p53 at Ser 15 is recognised as one of the central events in response to the ATM-Chk2 pathway induced double strand break repair [18]. Under conditions of sustained DNA damage expression of p53 Ser 15 phosphorylation was the most prominent in case of all the cells silenced for MDC1 expression as compared to the cells overexpressing MDC1. Also, p53 has been reported to escape E6 mediated degradation in cervical cancer cells after cisplatin treatment [19, 20]. Although, the exact mechanism for the decrease in p53 phosphorylation in combination with MDC1 overexpression still remains elusive but on the basis of our results, it could be interpreted that down regulating MDC1 expression in cervical cancer cells favoured p53 mediated apoptosis in response to cisplatin treatment.

### Discussion

Cervical carcinomas are often characterized by the abrogation of p53 tumor suppressor pathway mediated by HPV E6 and E7 oncogenes [21]. Additionally, HPV proteins are known to be responsible for activating the ATM-Chk2 pathway for aiding viral genome amplification [4]. Hence, regulation of ATM-Chk2 pathway can be a helpful approach towards addressing the spread of HPV infection in case of ATM proficient cells. Simultaneously, cisplatin has remained the first line of therapy for cervical cancer treatment and is reported to induce p53 mediated apoptosis [20]. The efficacy of cisplatin is limited by various factors including DNA repair mechanisms, which play a pivotal role in drug resistance [22]. Such factors together suggest the requirement for utilization of novel targeted therapies. We have shown that MDC1, a master regulator in the ATM-Chk2 pathway can be utilised for the sensitization of cervical cancer cells to chemotherapy. In the present study, we have evaluated the sensitivity to cisplatin in three high risk HPV positive cervical cancer cell lines with respect to the change in MDC1 expression. In our cisplatin treated cell lines, γ-H2AX accumulation was hindered in MDC1 silenced cells, resulting in defective foci formation in response to the DNA damage while the MDC1 overexpressing cells showed greater γ-H2AX accumulation, indicating an efficient DDR and cell survival [23].

Our results noticeably indicate that MDC1 knock down increases sensitivity to cisplatin in the cervical cancer cell lines while its overexpression facilitates cisplatin insensitivity in the cells. Investigation of p53 Ser 15 status led us to conclude that MDC1 through its interaction with p53 determines cell survival or apoptosis in response to genotoxic stress. MDC1 overexpression leads to a lower level of apoptosis following damage induction, whereas its downregulation leads to higher levels of apoptosis. The phosphorylation of Serine 15 residue on p53 protein can be mediated by both ATM and ATR protein kinases and is quite important for p53 activation by promoting its phosphorylation on additional serine residues which are necessary for its proper stabilization [24, 25].

Furthermore, Chk2, a protein kinase that acts downstream of ATM kinase, plays an important role in increasing intracellular p53 levels in response to DNA damage and a decline in its expression causes a defect in p53-mediated apoptosis [26]. In general, Chk2 phosphorylated by ATM at threonine 68 residue actuates p53 phosphorylation on serine 20, which interferes with interaction between p53 and Mdm2 and hence, increases p53 stability by preventing its ubiquitination in response to DNA damage [27]. Our results indicate that MDC1 expression modulation does influence the phosphorylation of p53 protein and hence, modifies the apoptotic response to cisplatin treatment accordingly. As a result, the anti-apoptotic nature of MDC1 observed so far because of the inhibition of p53 apoptotic activity could be due to a direct interaction and binding between the two proteins which might block the transactivation domain of p53 from getting phosphorylated by ATM kinase and Chk2 upon DNA damage [28].

## Limitations

Since cervical cancer cell lines are HPV positive monitoring E6 and E7 oncogene expression in our modified cell lines would have given greater insight as to increased cisplatin sensitivity in the MDC1 depleted cells. The investigation into p53 mediated apoptotic pathway becomes important factor in determining the fate of MDC1 silenced cells exposed to cisplatin. None the less the study clearly signifies that MDC1 in combination with cisplatin can prove to be an effective therapeutic approach for the treatment of cervical cancer patients.

## Supplementary information

**Additional file 1: Figure S1.** GIPZ MDC1 shRNA vector map (Dharamcon, G.E, USA) used to develop MDC1 shRNA expressing cervical cancer cell lines. It expresses microRNA-adapted shRNA based on miR-30 for specific gene silencing with minimal cytotoxicity. The vector additionally has GFP gene expressing a green fluorescence protein and puromycin as the selection marker.

**Additional file 2: Figure S2.** Vector map of pCDNA3 MDC1 full length construct was received as a kind gift from Prof. Michel Goldberg, Hebrew University, Israel. The vector has G418 as the selection marker.

**Additional file 3: Figure S3.** Uncropped images of the HeLa, SiHa and Caski cell lines modified for MDC1 expression and assessed with MDC1 primary antibody and beta actin (as loading control).

**Additional file 4: Figure S4.** Quantification of immunofluorescence images generated for HeLa, SiHa and Caski cells lines subjected to staining with anti-pγH2AX Ser 139 antibody following 2 h of cisplatin treatment (for pγH2AX Ser 139 treated cells in Fig. 5). The images were quantified using ImageJ software and analysed for the corrected total cell fluorescence (CTCF). CTCF = Integrated density − (Area of selected cell × Mean fluorescence of background readings).

## Data Availability

Not applicable.

## Abbreviations

CRT: Chemoradiotherapy
MDC1: Mediator of DNA damage checkpoint 1
DDR: DNA damage repair
ATM: Ataxia telangiectasia mutated
Chk2: Checkpoint kinase 2
γH2AX: Gamma histone 2A X
HR: Homologous recombination
NHEJ: Non-Homologous End Joining
