# Supplementary figures and images for: MDC1 depletion promotes cisplatin induced cell death in cervical cancer cells
Source: BMC Res Notes. 2020 Mar 11;13:146. doi: 10.1186/s13104-020-04996-5 (PMC7066845; doi:10.1186/s13104-020-04996-5)

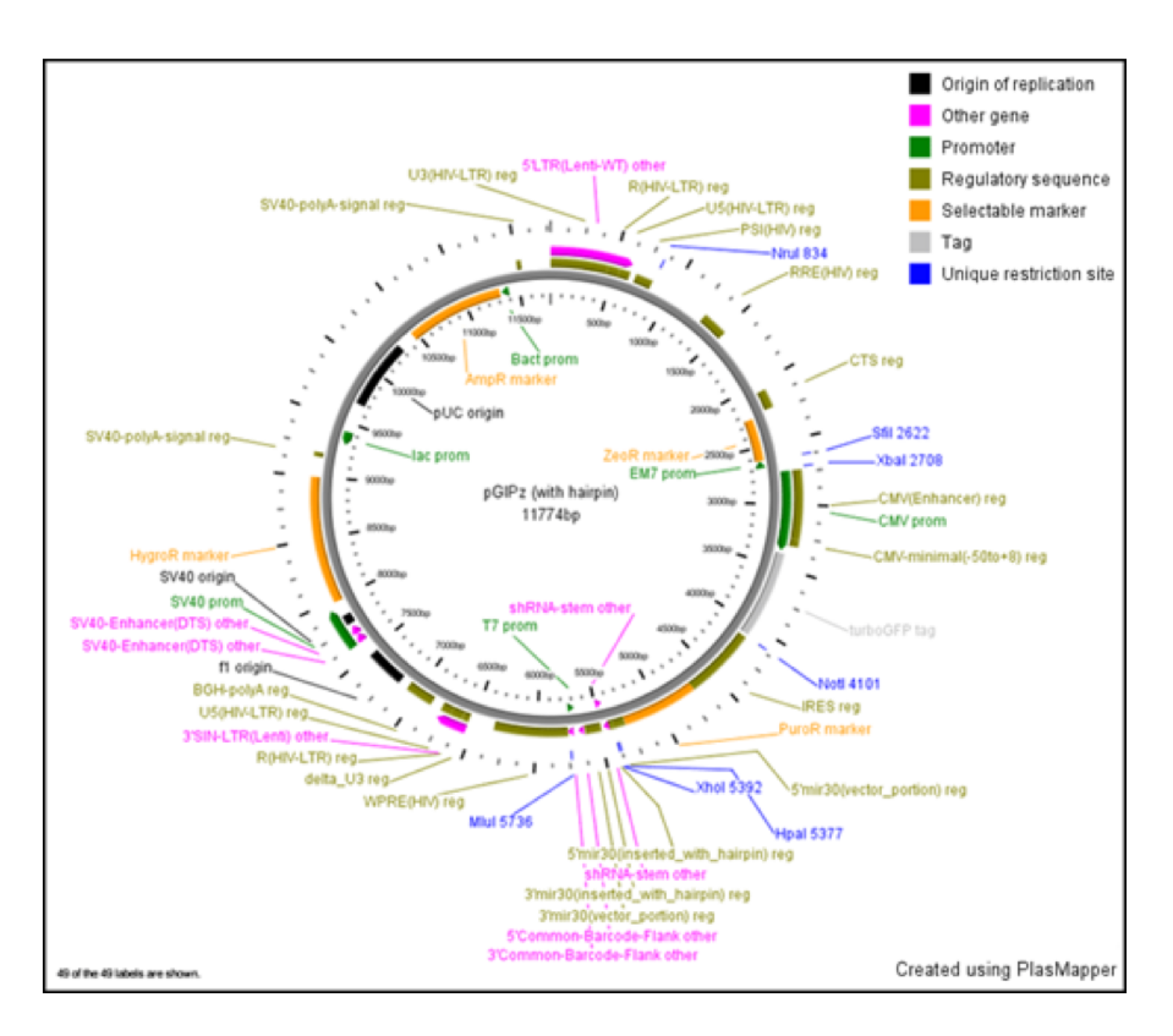

Supplement: Supplementary file 1 — Additional file 1: Figure S1. GIPZ MDC1 shRNA vector map (Dharamcon, G.E, USA) used to develop MDC1 shRNA expressing cervical cancer cell lines. It expresses microRNA-adapted shRNA based on miR-30 for specific gene silencing with minimal cytotoxicity. The vector additionally has GFP gene expressing a green fluorescence protein and puromycin as the selection marker. [file 13104_2020_4996_MOESM1_ESM.tiff]

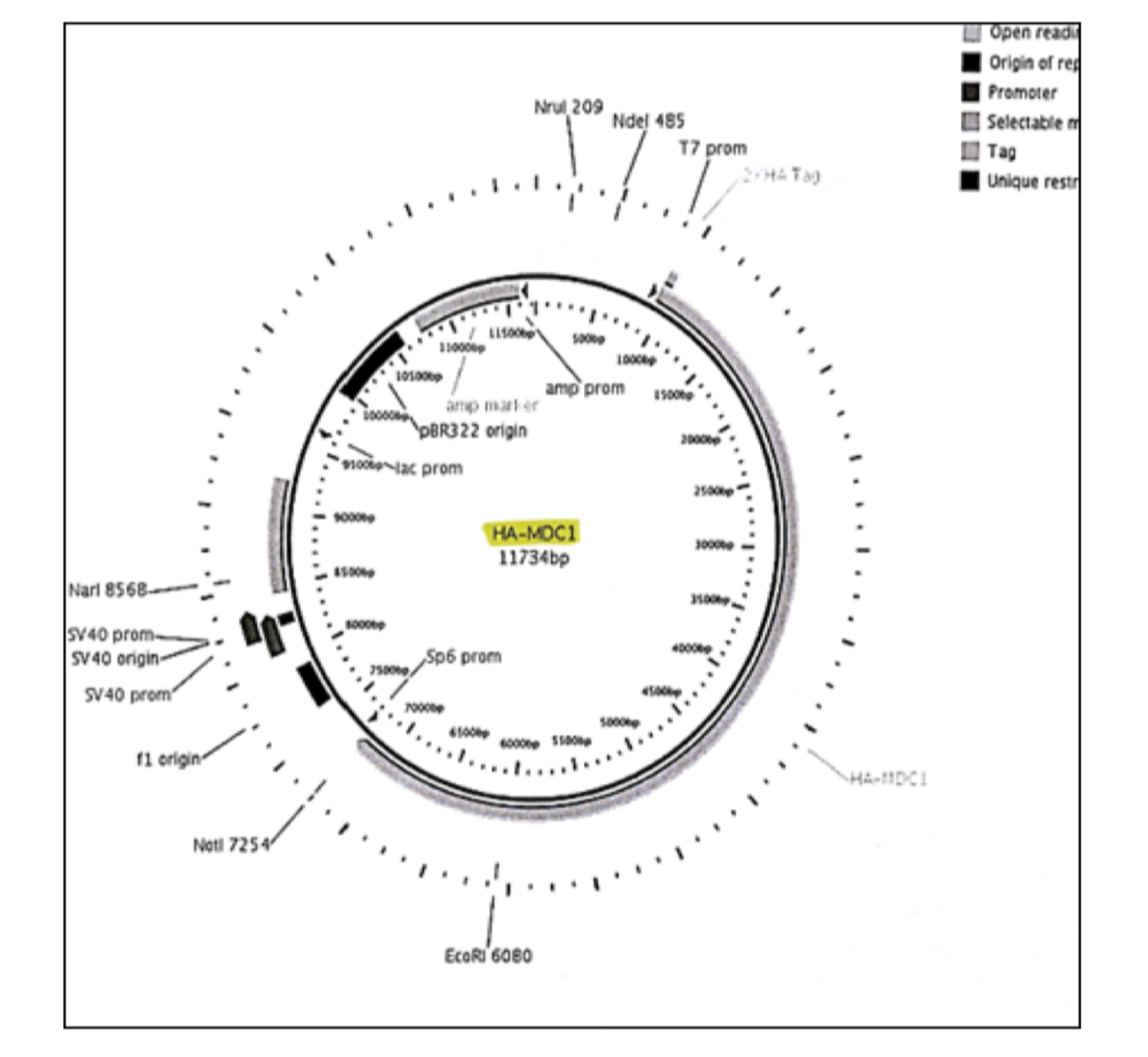

Supplement: Supplementary file 2 — Additional file 2: Figure S2. Vector map of pCDNA3 MDC1 full length construct was received as a kind gift from Prof. Michel Goldberg, Hebrew University, Israel. The vector has G418 as the selection marker. [file 13104_2020_4996_MOESM2_ESM.tiff]

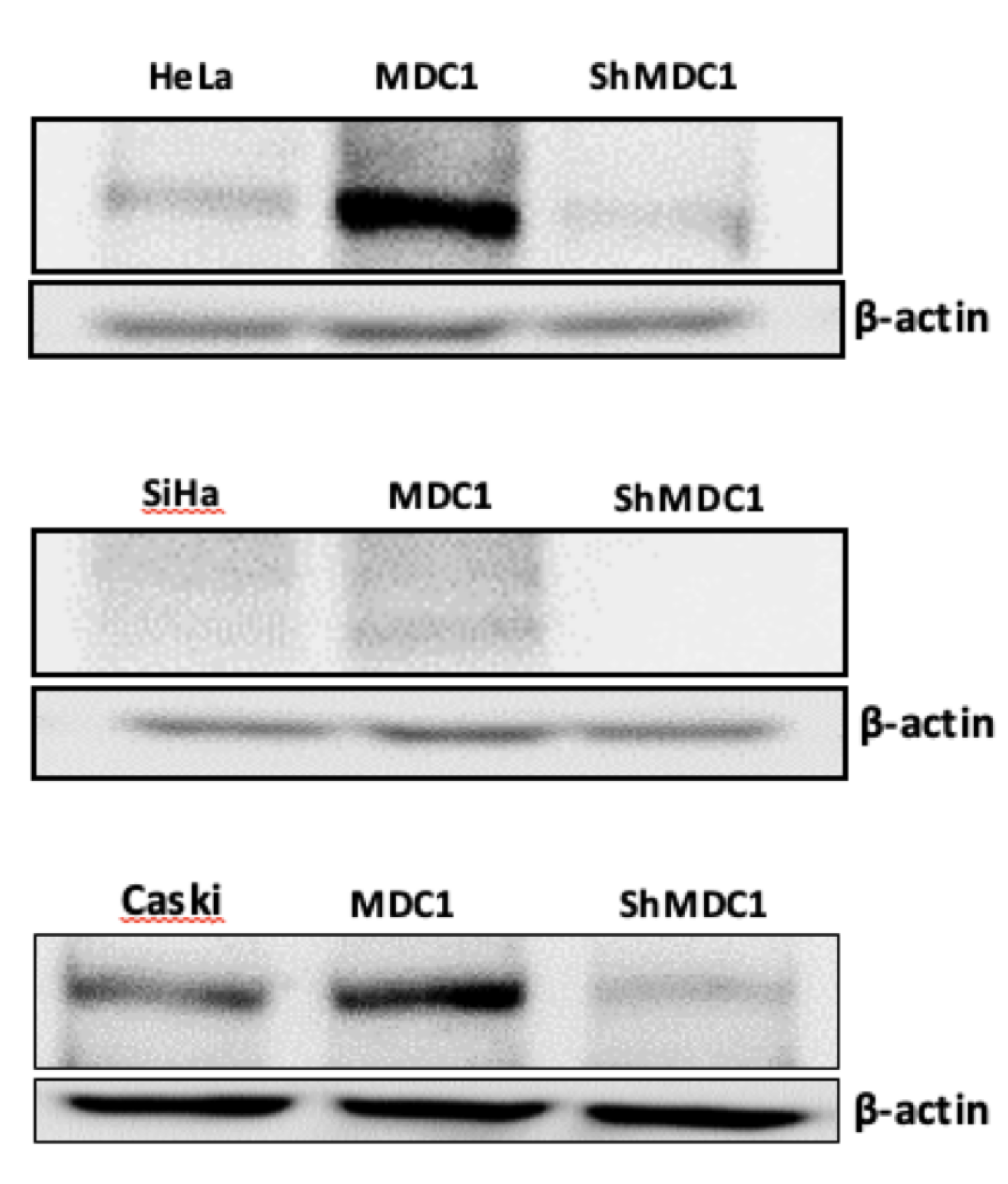

Supplement: Supplementary file 3 — Additional file 3: Figure S3. Uncropped images of the HeLa, SiHa and Caski cell lines modified for MDC1 expression and assessed with MDC1 primary antibody and beta actin (as loading control). [file 13104_2020_4996_MOESM3_ESM.tiff]

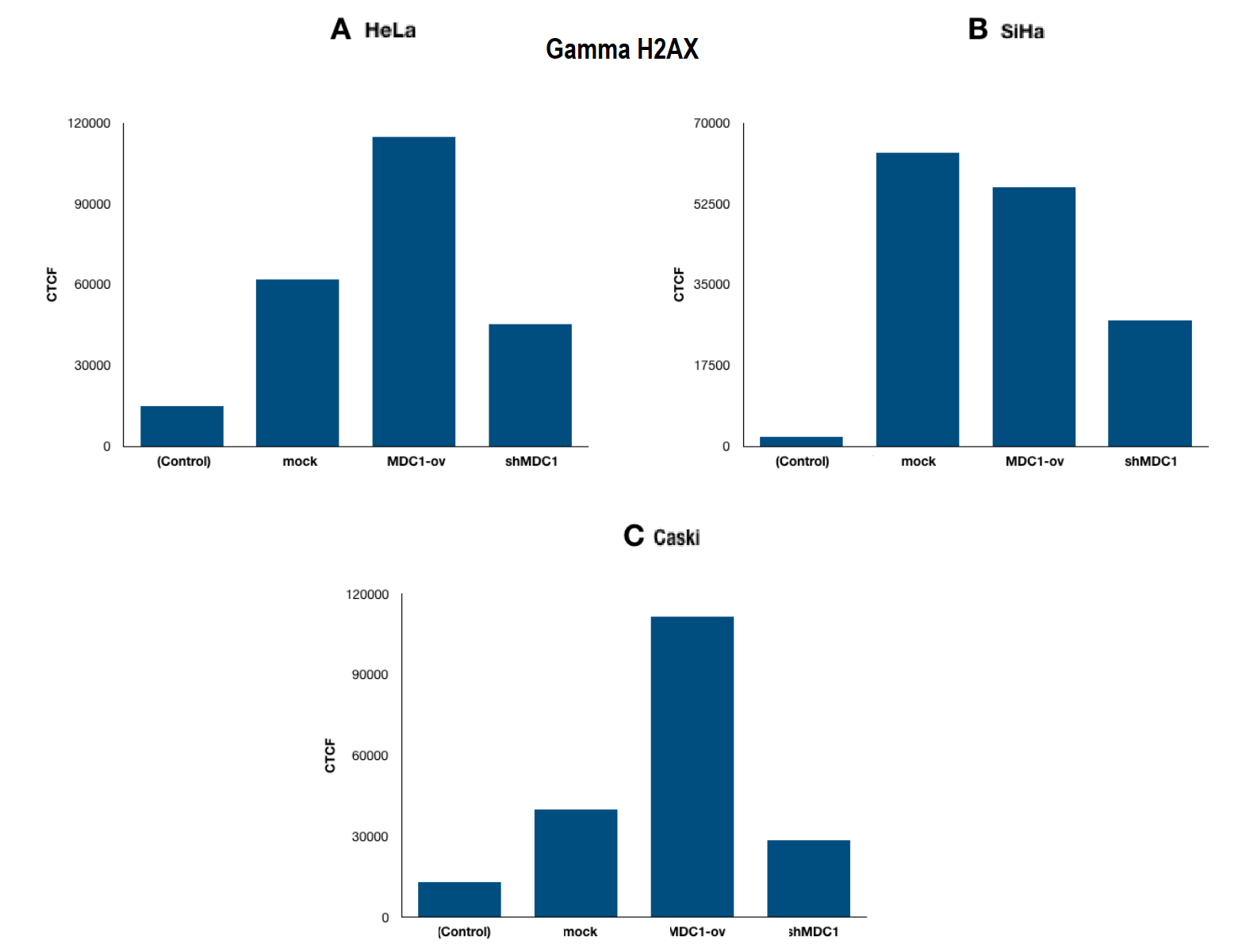

Supplement: Supplementary file 4 — Additional file 4: Figure S4. Quantification of immunofluorescence images generated for HeLa, SiHa and Caski cells lines subjected to staining with anti-pγH2AX Ser 139 antibody following 2 h of cisplatin treatment (for pγH2AX Ser 139 treated cells in Fig. 5). The images were quantified using ImageJ software and analysed for the corrected total cell fluorescence (CTCF). CTCF = Integrated density − (Area of selected cell × Mean fluorescence of background readings). [file 13104_2020_4996_MOESM4_ESM.tiff]
